# Analysis of changes in sodium and chloride ion transport in the skin

**DOI:** 10.1038/s41598-020-75275-3

**Published:** 2020-10-22

**Authors:** Iga Hołyńska-Iwan, Karolina Szewczyk-Golec

**Affiliations:** 1 Laboratory of Electrophysiology of Epithelial Tissue and Skin, Department of Pathobiochemistry and Clinical Chemistry, Faculty of Pharmacy, Collegium Medicum in Bydgoszcz Nicolaus Copernicus University in Torun, M. Skłodowskiej-Curie 9, 85-094 Bydgoszcz, Poland; 2 Department of Medical Biology and Biochemistry, Faculty of Medicine, Collegium Medicum in Bydgoszcz Nicolaus Copernicus University in Torun, Bydgoszcz, Poland

**Keywords:** Physiology, Medical research

## Abstract

The measurement of electric potential and resistance reflect the transport of sodium and chloride ions which take place in keratinocytes and is associated with skin response to stimuli arising from external and internal environment. The aim of the study was to assess changes in electrical resistance and the transport of chloride and sodium ions, under iso-osmotic conditions and following the use of inhibitors affecting these ions’ transport, namely amiloride (A) and bumetanide (B). The experiment was performed on 104 fragments of rabbit skin, divided into three groups: control (n = 35), A—inhibited sodium transport (n = 33) and B—inhibited chloride transport (n = 36). Measurement of electrical resistance (R) and electrical potential (PD) confirmed tissue viability during the experiment, no statistically significant differences in relation to control conditions were noted. The minimal and maximal PD measured during stimulation confirmed the repeatability of the recorded reactions to the mechanical and mechanical–chemical stimulus for all examined groups. Measurement of PD during stimulation showed differences in the transport of sodium and chloride ions in each of the analyzed groups relative to the control. The statistical analysis of the PD measured in stationary conditions and during mechanical and/or mechanical–chemical stimulation proved that changes in sodium and chloride ion transport constitute the physiological response of keratinocytes to changes in environmental conditions for all applied experimental conditions. Assessment of transdermal ion transport changes may be a useful tool for assessing the skin condition with tendency to pain hyperactivity and hypersensitivity to xenobiotics.

## Introduction

Analysis of changes in the electrophysiological parameters of the skin in mammals in association with disrupted transmembrane ion transport remains a common research topic. Assessment of the transport of sodium and chloride ions offers the possibility to observe both physiological responses and those altered by exogenous substances used experimentally within a tissue structure with preserved layers, reactivity and sensitivity to stimuli^1–4^. Epithelial sodium channels (ENaC) present on keratinocytes are involved in the transport of water into and out of the cells, modifying osmolality both inside the cell and in the adjacent space^3–6^. Every opening of an ENaC is associated with the influx of sodium ions followed by water molecules from the extracellular space. Closing of an ENaC results in the efflux of water from the cell to balance osmolality. Substances altering the functioning of ENaCs lead to changes in the hydration of keratinocytes and the surrounding environment^3–5^. Secretion of chloride ions by the keratinocyte membrane occurs through CFTR (cystic fibrosis transmembrane regulator) channels^7^ and other chloride channels, e.g., CLCA (chloride channel accessory)^8^. Moreover, the presence of CFTR channels has been demonstrated on cells lining sweat ducts^9^. A change in the functioning of chloride channels on keratinocytes and/or sweat duct cells may cause water flow and dehydration or overhydration of cells constituting the appropriate skin layer or the surrounding environment^6,9,10^. Moreover, CFTR channels act as cell regulators that affect, e.g., the functioning of ENaCs^7–9^. Changes in the functioning of sodium and/or chloride channels can underlie problems with regeneration and healing^10^, onset of hypersensitivity and/or allergies^3,5^, atopic dermatitis^8^ and hypersensitivity to pain^11,12^. Few studies on this subject have been published to date^3,4,7–10^.

The aim of the study was to assess changes in electrical resistance and the transport of chloride and sodium ions, measured as transepithelial electrical potential in fragments of the skin of experimental animals, under iso-osmotic conditions and following the use of inhibitors of these ions’ transport.

## Results

Transmembrane PD measured in stationary conditions for tissues incubated in RH was − 0.25 mV, while the median measurements for solutions A and B were − 0.31 and − 0.23 mV, respectively. No statistically significant differences were demonstrated between the measured parameters (Table 1, see Supplementary Figs. 1S and 2S online). This result indicates that incubation of the tissues in conditions inhibiting the transport of one of the ion types did not cause a significant change in the generation of electric field by the tissues.

**Table 1:** Values of analyzed parameters of skin specimens.

Incubation/stimulation solution	Stationary conditions		Stimulation conditions (15 s)		Wilcoxon test (*p*)		
Parameter	R (Ω*cm^2^)	PD (mV)	PDmin (mV)	PDmax (mV)	PD vs. PDmin	PD vs. PDmax	PDmin vs. PDmax
**RH (n = 70)**							
Median	844	− 0.25	− 0.15	0.15	0.253	*0*	*0*
Lower quartile	438	− 0.66	− 0.95	− 0.12			
Upper quartile	1360	0	0	0.7			
**A (n = 66)**							
Median	1024	− 0.31	− 0.45	0	*0*	*0*	*0*
Lower quartile	740	− 1.04	− 1.27	− 0.94			
Upper quartile	1563	0.18	− 0.09	0.69			
**B (n = 72)**							
Median	894	− 0.23	− 1.16	0	*0*	*0*	*0*
Lower quartile	610	− 1.9	− 2.32	− 1.77			
Upper quartile	1136	0.12	0	0.37			
**Mann–Whitney test (p)**							
RH vs. A	0.013	0.585	*0.025*	*0.028*			
RH vs. B	0.929	0.238	*0.001*	*0.001*			
A vs. B	*0.007*	0.142	0.062	0.119			

*RH* isoosmotic Ringer solution, *A* inhibited sodium transport by amiloride (0.1 mM), *B* inhibited chloride transport by bumetanide (0.1 mM), *PD* transepithelial potential difference of epithelial skin surface measured in stationary conditions (mV), *PDmin* minimal transepithelial potential difference during 15 s stimulation of epithelial skin surface (mV), *PDmax* maximal transepithelial potential difference during 15 s stimulation of epithelial skin surface (mV), *R* resistance measured in stationary conditions (Ω*cm^2^), italic values indicate a level of significance *p* < 0.05.

**Figure 1:**
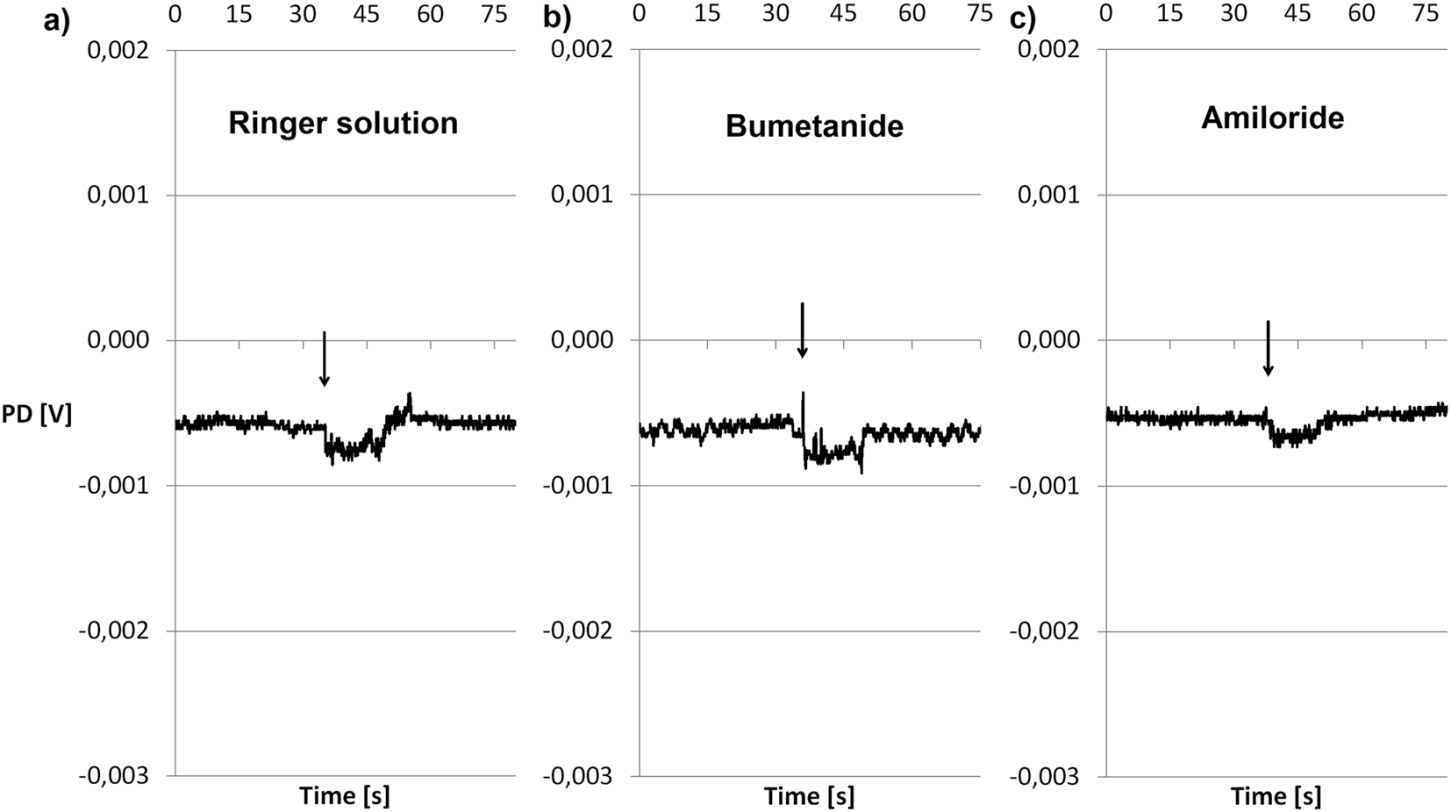
Voltage of tissue samples during stimulation with the following solutions. (**a**) Ringer solution as an incubation and bathing fluid, as well as a mechanical stimulation—the control group. (**b**) Bumetanide solution as an incubation and bathing fluid, as well as a mechanical–chemical stimulation—the group of inhibited chloride ion transport. (**c**) Amiloride solution as an incubation and bathing fluid, as well as a mechanical–chemical stimulation—the group of inhibited sodium ion transport. Time-courses of single experiments are presented, showing the most frequently observed electrophysiological responses to stimulation. The arrows indicate mechanical (Ringer solution) or mechanical–chemical stimulation (bumetanide or amiloride).

Mechanical (RH) and mechanical–chemical (A, B) stimulation resulted in repeated changes in ion transport measured as PDmin and PDmax during the 15-s stimulation (Fig. 1). As expected in the case of electrophysiological studies of living skin fragments, different patterns of response to the stimuli were observed. However, for each stimulation (RH, A, B), the statistical analysis indicated the hyperpolarization reaction as significantly predominant (Table 1). Depolarization was found in 24% of the response to stimulation with RH, 20% of the response to A and 10% of the response to B. Incubation in RH induced a minimum potential of − 0.15 mV. Specimens stimulated with solution A had a PDmin of − 0.45 mV, while those stimulated with solution B had a PDmin of − 1.16 mV. PDmax was 0.15 mV for the control specimens and 0 mV for those incubated with either inhibitor. All measured PDmax and PDmin values were different in a statistically significant manner from the potential values in stationary conditions for each investigated group (Table 1, Wilcoxon test). Tissues incubated in the solution of chloride ion transport inhibitor demonstrated the highest potential measured during stimulation. Both PDmin and PDmax for incubations in solution B were different in a statistically significant manner from the potentials measured in solution A. No differences were noted for solutions A and B in relation to the control (RH) (Table 1, Mann–Whitney test).

The median R value of the examined skin fragments incubated in RH was 844 Ω*cm^2^. Tissue samples incubated in solution A had R = 1024 Ω*cm^2^, while those treated with solution B had R = 894 Ω*cm^2^. The R values measured in the solutions inhibiting ion transport were statistically different from each other (*p* = 0.007), but did not demonstrate differences in relation to the control (Table 1).

The described electrophysiological parameters were measured twice. There were no statistically significant differences between the PD and R values recorded in the same conditions.

## Discussion

The Ussing chamber was originally used to analyze epithelium-lined organs, such as the airways^13,14^ or the digestive tract^15^, and to elucidate the pathomechanisms of diseases associated with disrupted function of ion transporters and/or channels^7,13–15^. The modification of the Ussing chamber involved positioning of the analyzed specimen horizontally and its mechanical stimulation with a nozzle located 10 mm from the surface of the analyzed tissue^14,15^. The proposed experimental model for the assessment of electrophysiological parameters in the modified Ussing chamber is based on an analysis of full-thickness skin fragments with preserved layered structure and nerve endings^1,2^. It also allows assessing the functioning of transporters and channels maintaining a constant flow of ions in conditions both similar to physiological or altered^1,2,16^. Tissue samples taken from experimental animals have preserved nerve endings, layered structure and the ability of cells to respond to incubation with the selected substances^2^. Current studies put emphasis on the assessment of changes in the microenvironment of cells, as well as those occurring intracellularly^8,10–12,17,18^. Any disruption of homeostasis may result in a response of the body, including changes in the composition of the extracellular matrix and in the quantity or differentiation of cells^4,11,17^. The proposed model reflects changes in the transport of ions in cells arranged in layers, as well as in intercellular spaces.

Electrical resistance was calculated based on the change in potential after passing electric current of a constant intensity through the tissue^2^. Therefore, changes in resistance are due to changes in ion transport, including the functioning of channels and transporters, as well as due to the structure and compactness of the analyzed tissues, including the degree of cell adhesion, tissue damage and deformation^1,2,16^. The values of resistance obtained for tissues incubated in an iso-osmotic environment after adding inhibitors of sodium and chloride ion transport indicate that full vitality and integrity along with tight junctions between cells were preserved^1,16^. Obtained resistance values proved that all specimens were alive during experiment and tested substances did not affect their vitality and tightness. Resistance values obtained for tissue fragments treated with sodium and chloride ion transport inhibitors were different from each other in a statistically significant manner, which may indicate a change in the permeability of cells to each ion type (see Supplementary Fig. 1S online). However, they were not different from those obtained for the control specimens, as the use of iso-osmotic Ringer’s solution and ion transport inhibitors did not cause changes in cell-to-cell and cell-to-matrix adhesion and/or in the permeability of the tested specimens to ions^2,9^. Similar research was described by Barker et al.^19^, who studied electrophysiological parameters of glabrous and gland-free skin parts of guinea pig. Interestingly, they found a resistance values significantly higher than the ones reported in the present study. It should be noted that the authors used different measurement techniques. First of all, they made measurements in skin incisions of live animals, both in hairless and hairy skin parts. In the case of hairy skin, they obtained significantly lower resistance values compared to the hairless parts, which results are similar to those detected in the present study. In our experiment, a model of hairy, undamaged skin was used and, what is even more important, the electrophysiological parameters were measured through the entire skin fragments, not across the epidermis, as in the Barker et al. study^19^.

The lack of differences in potential measured in stationary conditions constitutes evidence of the preserved functioning of ion pumps, channels and co-transporters in all studied specimen groups (Table 1, see Supplementary Fig. 1S online). Transmembrane potential was stable and depended on the flow of sodium and chloride ions in tissues incubated in RH, increased efflux of chloride ions in tissues treated with amiloride solution and increased influx of sodium ions in tissues treated with bumetanide solution. Stable PD proved that no morfological changes occured on specimen surface and the activity of the cells were preserved. It can be presumed that the tissues managed to adapt to the changed environmental conditions, similarly as has been observed for the airways^13,14^ and the digestive tract^15^.

The electric potential on the surface of hairless mice skin in organ culture was the subject of the study by Denda et al.^6^. In their experiment, the potential values were lower (approximately − 3 mV) than those measured in the present study (− 0.31 to − 0.23 mV). Also in the study by Barker et al.^19^, the values of the potential measured in the epidermis of the guinea pig’s abdominal skin were significantly lower, amounting to about − 6 mV. The differences between the methods used in the experiments may explain such substantial discrepancies in potential values measured. In the Barker et al.^19^ experiment, live animals with skin incisions were examined and transepithelial potentials were measured using appropriate electrodes in the Ringer solution at pH 5.8. In Denda et al.^6^ study, skin samples were placed in Dulbecco's modified Eagle medium (DMEM), used for cell culture, and the experiment was conducted at 37 °C. Thus, in both studies the cellular metabolism rate in the tested skin parts was high. It was proven in the Denda et al.^6^ study that the value of the potential is greatly influenced by the intact production of ATP. Due to the disruption of mitochondrial function, the transepithelial potential in their study increased to about − 0.8 mV. In the present experiment, the examined skin samples were well-preserved and alive, but their metabolic rate was reduced due to the lack of an external source of energy substrates and the use of a temperature lower than optimal for metabolic processes. Thus, a limited amount of ATP was avalaible for ion transport. Additionally, the rabbit skin, used in our experiment, has a different thickness, as well as nervous, hormonal and immunological regulation compared to mouse or guinea pig skin^1^, which could also affect the results.

In the present study the use of a 15-s stimulus caused repeatable changes in the transport of ions measured as minimum and maximum potential (Fig. 1, Table 1, see Supplementary Fig. 2S online).

The applied mechanical and mechanical–chemical stimulations caused different directions of the electrophysiological response in the examined skin fragments, which could be explained by diverse reactions in the transport of sodium, chloride and potassium ions. The most frequently observed reaction pattern was hyperpolarization, which could be associated with the predominance of the sodium ion absorption from the tissue surface and/or chloride ion secretion. However, a depolarization reaction was observed in some skin samples, most likely due to the inhibition of sodium ion absorption or chloride ion secretion and the initiation of potassium ion uptake from the cell surface. Such differences in responses may result from specific characteristics of the tested tissue samples, such as the level of epithelial hydration, the presence of minimal scarring and/or local dermal inflammation, as well as the chemical composition of skin extracellular matrix, including the presence of specific proteins, lipid barrier components or inflammatory cytokines^3,4,10^. However, despite the diversity of the observed responses, hyperpolarization was estimated as a statistically significant direction of changes in the transepithelial potential for each type of stimulation.

Both PDmin and PDmax measured with inhibited transport of chloride and sodium ions were different in a statistically significant manner from the control. Hence, the flow of fluid resulted in an increased efflux of chloride ions with inhibited transport of sodium ions and an increased influx of sodium ions with inhibited transport of chloride ions. However, these responses were more intense from those observed for mechanical stimulation only. The transport of sodium ions during incubation and stimulation with bumetanide was intensified. This phenomenon can be explained by an increased sensitivity of cells to factors modulating the transport of sodium ions compared to chloride ions^9^. ENaCs are present in large numbers on keratinocytes and take part in the regulation of cell hydration^3^, efflux of small-molecule substances^11^ and proinflammatory factors^3^, as well as initiation of cell differentiation and migration^5^. It has been shown that even minimal changes in the transport of sodium ions in the skin may be associated with the onset of inflammation and migration of immunocompetent cells^3^, onset of hypersensitivity reaction and/or allergies^17^, hypersensitivity to pain^11,12^, slowed regeneration process^5^ and exacerbations of skin lesions in many diseases^17^. The presence of substances altering sodium transport may be of importance in changing the approach to treating difficult-to-heal wounds and ulcerations and in an increased incidence of hypersensitivity reactions and allergies, in particular to drugs^17^.

In the experiment, the use of amiloride, an inhibitor of sodium transport, caused an increased efflux of chloride ions, and the response to the flushing of the external surface of the skin was the most intense. Median PDmin and PDmax were significantly higher than those measured during RH stimulation (Table 1, Mann–Whitney test, see Supplementary Fig. 2S online). Preserved physiological efflux of chloride ions is important for the proper flow of water and dissolved substances between the layers of the skin^10^, as well as for the processes of perspiration, cooling of the skin surface and excretion of metabolites or xenobiotics^7,8^. Intensification of chloride transport can also contribute to facilitated cell migration and, consequently, can initiate the processes of healing and regeneration^10^. However, alteration of the functioning of the CFTR channel may contribute to the observed changes in drug metabolism, as well as occurrence of adverse reactions or hypersensitivity to drugs or light, which is of particular importance for the treatment of patients with cystic fibrosis and difficult-to-heal wounds^7,18^.

The effect of amiloride on the skin electric potential was investigated by Barker et al.^19^, who observed significant decreases in the potential across the glabrous and gland-free guinea pig epidermis when exposed to a 5 mM amiloride solution for 2 min. It should be noted that the skin studied in their experiment was sweatless. It could therefore be assumed that, contrary to our study, the modifications in the chloride ion transport were not involved in the skin response to the amiloride exposure. This is why the results obtained by Baker et al.^19^ were analogous to the electrophysiological properties of amphibian skin. Denda et al.^6^, in their experiment on electrophysiological parameters of hairless mice skin, also investigated the effect of sodium transport inhibition on the skin electric potential. Tetrodotoxin, an inhibitor of voltage-gated sodium channels (NaV channels) characteristic of excitable cells^20^, was used in their study. ENaC channels, which are amiloride-sensitive, are not inhibited by tetrodotoxin. The significant decreases in the transcutaneous potential after 1 h of incubation in a 50 µM tetrodotoxin solution resulted from the inhibition of NaV channels, which were not examined in the present study. It is worth adding that Denga et al.^6^ used a much longer incubation time in the inhibitor solution. What is also important, the skin of a rabbit and a rodent has different characteristics^21^. All of these factors may contribute to the discrepancies between the results of both studies.

## Conclusions

The study demonstrated that the use of inhibitors of sodium and chloride ion transport causes changes in electrical resistance and transmembrane potential which can be measured using a modified Ussing chamber. It has been shown that measuring the electrophysiological parameters of the skin in mammals can be a valuable tool to assess homeostasis in the skin tissue. Use of simple inhibitors of sodium or chloride ion transport may be the basis for establishing which ion transport system has been altered by the analyzed factor. It seems that determination, which of the medicines, xenobiotics or toxins used disturb this delicate mechanisms and how it happens, may be the background for drawing conclusions regarding the observed symptoms, proposed treatment or therapeutic approach in many diseases occurring with skin disruption.

## Materials and methods

### Study design

Isolated skin specimens (n = 104) were derived from five adult, albino, New Zealand rabbits, 2–3 months old, of both sexes, with a body weight of 3.5–4.0 kg. The animals were housed in disposable cages and allocated two rabbits per cage, in the 12/12 light/dark cycle, with water and food available ad libitum. The rabbits were killed by asphyxiation using CO_2_ (approx. 60% in the inhaled air). After animal death, skin samples from the abdomen were taken, with hair shaved mechanically. Subsequently, the skin was severed, and the membranous part, muscle, fat and vessels were discarded. The skin specimens were collected from dead animals. The presented experiments did not include living animals and according to the European Union law did not require bioethical committee agreement. The animals were kept and killed in accordance with the guidelines and regulations of Nicolaus Copernicus University following the Polish Animal Protection Act and the European Directive on the Protection of Animals Used for Scientific Purposes (2010/63/EU) by qualified personnel with all certificates for killing animals in the laboratory (No 14/2016 valid from 10/20/2016 to 10/11/2021).

The skin samples were cut into 1 cm^2^ pieces and randomly divided into three groups to be incubated for 30 min at room temperature in three different solutions.

1. Control (n = 35): specimens incubated in Ringer’s solution (RH),
2. Inhibited sodium transport (n = 33): specimens incubated in amiloride solution (A),
3. Inhibited chloride transport (n = 36): specimens incubated in bumetanide solution (B).

Subsequently, the specimens were horizontally mounted in a modified Ussing chamber. The modification allowed mechanical stimulation of the stratum corneum of the skin with fluid using a peristaltic pump with a flow of 0.06 ml/s (1 ml/15 s). The stimulation nozzle was placed at a distance of 4–6 mm from the tested tissue surface. Below the level of the stimulation nozzle, on the other side of the chamber, there were vent holes allowing the excess liquid to flow freely, which eliminated the pressure difference. The fluid administration in a manner imitating drops falling on the skin surface, was considered a mechanical stimulation of the tested sample. Constant current electrodes and the measuring electrode were placed at a distance of 10 mm from the tested skin surface. Scheme of the measuring system is avalaible in Supplementary Information (see Supplementary Fig. 3S online).

**Figure 2:**
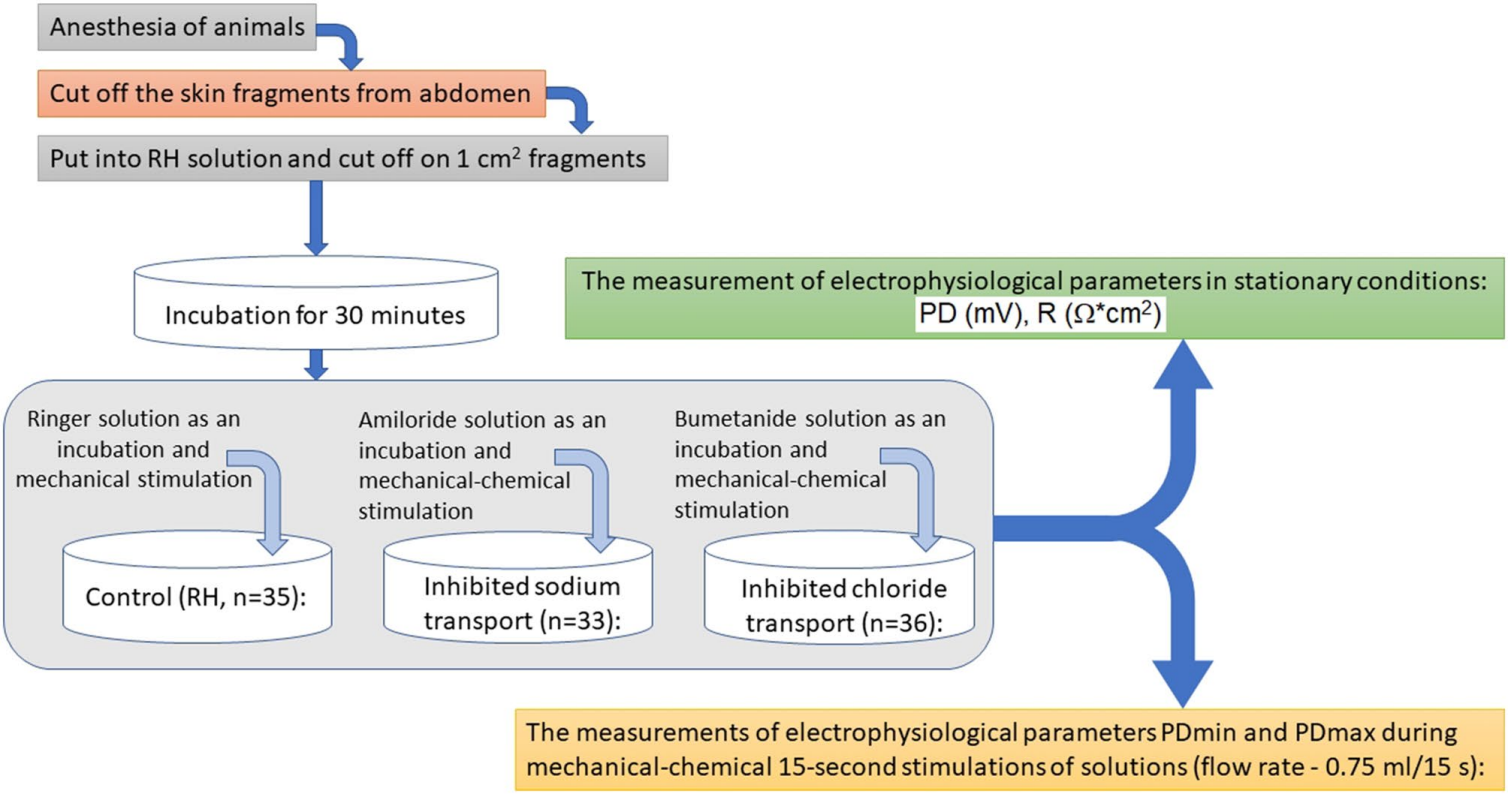
Study design. *n* number of skin specimens, *RH* Ringer solution, *PD* transepithelial potential difference of skin surface (mV) in stationary conditions, *PDmin* minimal transepithelial potential difference during 15 s stimulation of skin surface (mV), *PDmax* maximal transepithelial potential difference during 15 s stimulation of skin surface (mV), *R* resistance (Ω*cm^2^).

After the electrophysiological parameters were stabilized for all fragments, series of mechanical (RH) and mechanical–chemical (A, B) stimulation were applied (Fig. 2).

The experiments consisted of measuring twice the following parameters:

- transepithelial potential difference—changes in transepithelial electrical potential in stationary conditions (PD, mV),
- minimum and maximum transepithelial electrical potential difference during 15-s stimulation (PDmin, PDmax, mV),
- transepithelial electrical resistance measured in stationary conditions (R, Ω*cm^2^).

PD was recorded continuously, while R was determined by stimulating the tissue with a current intensity of ± 10 µA. Subsequently, the corresponding voltage change was measured, and resistance was counted according to Ohm's law.

### Chemicals and solutions

The following chemicals and solutions were used in the experiment:

1. RH—Ringer solution, a basic solution with iso-osmotic properties and pH 7.4. Composition: Cl^−^ 160.8 mM; Na^+^ 147.2 mM; K^+^ 4.0 mM; Mg^2+^ 2.6 mM; Ca^2+^ 2.2 mM; HEPES 10.0 mM (4-(2-hydroxyethyl)piperazine-1-ethanosulfonic acid, 238.30 g/mol);
2. Amiloride (A)—used as an inhibitor of transepithelial transport of sodium ions, in a concentration in 0.1 mM solution of amidynoamide acid, 3,5-diamino-6-chloro-2-carboxylic acid (266.09 g/mol), dissolved and diluted in RH.
3. Bumetanide (B)—used as an inhibitor of transepithelial transport of chloride ions, in a concentration in 0.1 mM solution of 3-butylamino-4-phenoxy-5-sulfamoylbenzoic acid (364.42 g/mol), dissolved in 0.1% DMSO (dimethyl sulfoxide) and diluted in RH.

Reagents: amiloride, bumetanide, DMSO and HEPES were purchased from Sigma-Aldrich (USA). Mineral compounds: KCl, NaCl, CaCl_2_, MgCl_2_ were purchased from POCH (Poland).

### Data analysis

Data were recorded on an experimental apparatus EVC 4000 (WPI, USA), connected to the data acquisition system MP 150 which transferred the obtained data to the computer data acquisition software AcqKnowledge 3.8.1 (Biopac Systems, Inc., USA).

Results were presented as median and summarized in tables and graphs. Statistical analysis was conducted in the Statistica 11.00 software (StatSoft, Inc.). The Wilcoxon test was used to compare data from the same incubation conditions with the statistical significance level at *p* < 0.05. The Mann–Whitney test was used to detect significant differences (*p* < 0.05) for the different experimental conditions in the examined groups of tissue samples.

### Ethical approval

No experiments involving human participants were performed in the study. The present experiment did not include living animals and according to the Polish and European Union law, the bioethical committee agreement was not required. Animal care was in accordance with the guidelines and regulations as stipulated by the Polish Animal Protection Act and the European Directive on the Protection of Animals Used for Scientific Purposes (2010/63/EU). All applicable institutional and national guidelines for the care and use of animals were followed.

## Supplementary information

Supplementary Figures.

## Abbreviations

A: Amiloride solution (0.1 mM)
B: Bumetanide solution (0.1 mM)
CFTR: Cystic fibrosis transmembrane regulator
DMEM: Dulbecco’s modified Eagle medium
ENaC: Epithelial sodium channel
NaV: Voltage-gated sodium channel
PD: Transepithelial potential difference
PDmin: Minimum transepithelial potential difference during 15 s stimulation
PDmax: Maximum transepithelial potential difference during 15 s stimulation
R: Transepithelial electrical resistance
RH: Isoosmotic Ringer solution
