# Supplementary material for: Analysis of changes in sodium and chloride ion transport in the skin
Source: Sci Rep. 2020 Oct 22;10:18094. doi: 10.1038/s41598-020-75275-3 (PMC7581804; doi:10.1038/s41598-020-75275-3)
Supplement: Supplementary file 1 — Supplementary Figures. [file 41598_2020_75275_MOESM1_ESM.pdf]

Title: Analysis of changes in sodium and chloride ion transport in the skin as a valuable tool to assess the pathomechanism of skin disruption.

Iga Hołyńska-Iwan, Ph.D. [1*], Karolina Szewczyk-Golec, Ph. D. [2]

[1] Laboratory of Electrophysiology of Epithelial Tissue and Skin, Department of Pathobiochemistry and Clinical Chemistry, Faculty of Pharmacy, Collegium Medicum in Bydgoszcz Nicolaus Copernicus University in Torun, Poland

[2] Department of Medical Biology and Biochemistry, Faculty of Medicine, Collegium Medicum in Bydgoszcz Nicolaus Copernicus University in Torun, Poland

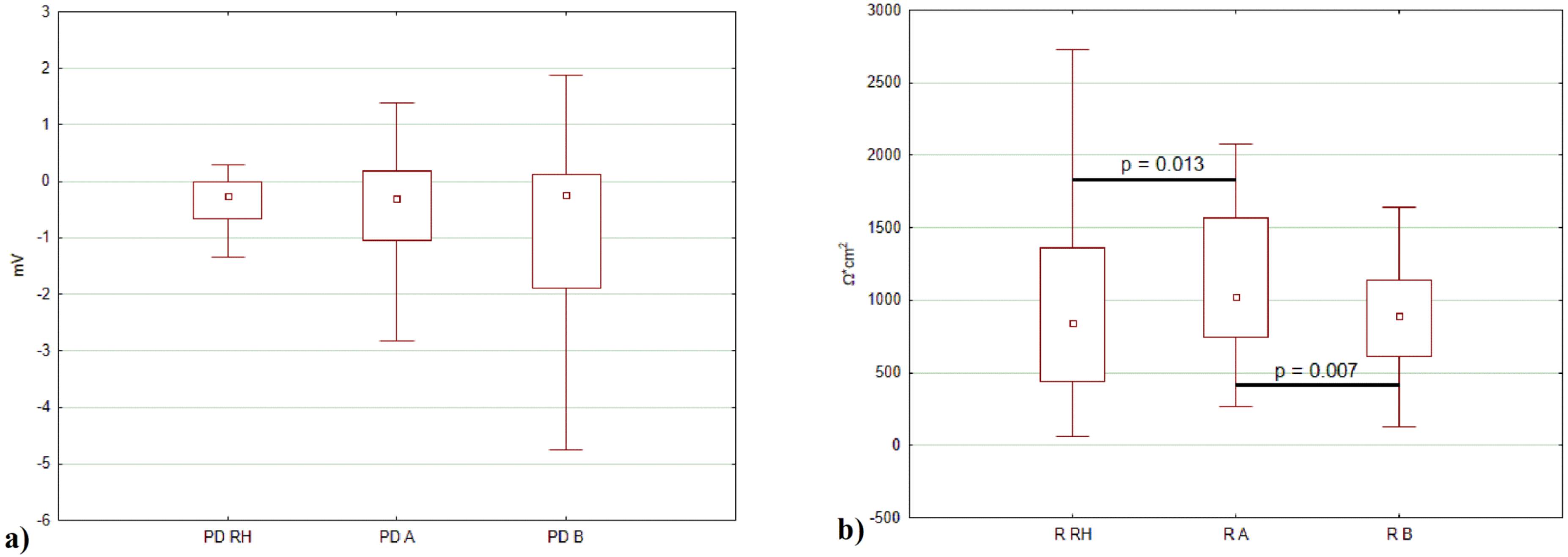

Figure 1S. The comparison of the transepithelial electric potential (PD, mV) and transepithelial resistance (R, $\Omega*cm^2$) measured in stationary conditions of the isolated rabbit skin specimens, incubated in: Ringer solution (**RH**), amiloride (**A**, 0.1 mM) and bumetanide (**B**, 0.1 mM):

a) PD measured before mechanical-chemical stimulations,

b) R measured after mechanical-chemical stimulations.

The values of medians and lower and upper quartiles are given. The P value < 0.05 were considered as statistically significant.

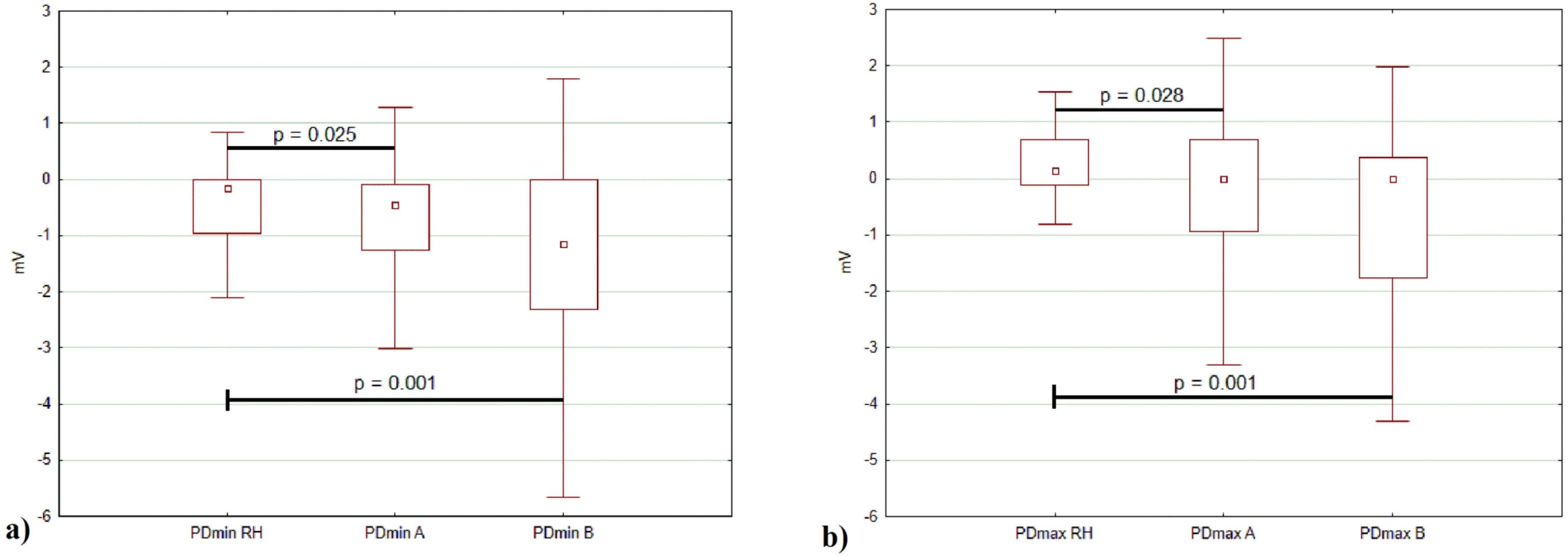

Figure 2S. The comparison of the minimal (PDmin, mV) and maximal (PDmax, mV) transepithelial electric potential measured in 15s mechanical-chemical stimulation of the isolated rabbit skin specimens, incubated and stimulated by: Ringer solution (**RH**), and amiloride (**A**, 0.1 mM) solution and bumetanide (**B**, 0.1 mM) solution:

a) PDmin measured during stimulation by RH, A and B solutions,

b) PDmax measured during stimulation by RH, A and B solutions.

The values of medians and lower and upper quartiles are given. The P value < 0.05 were considered as statistically significant.

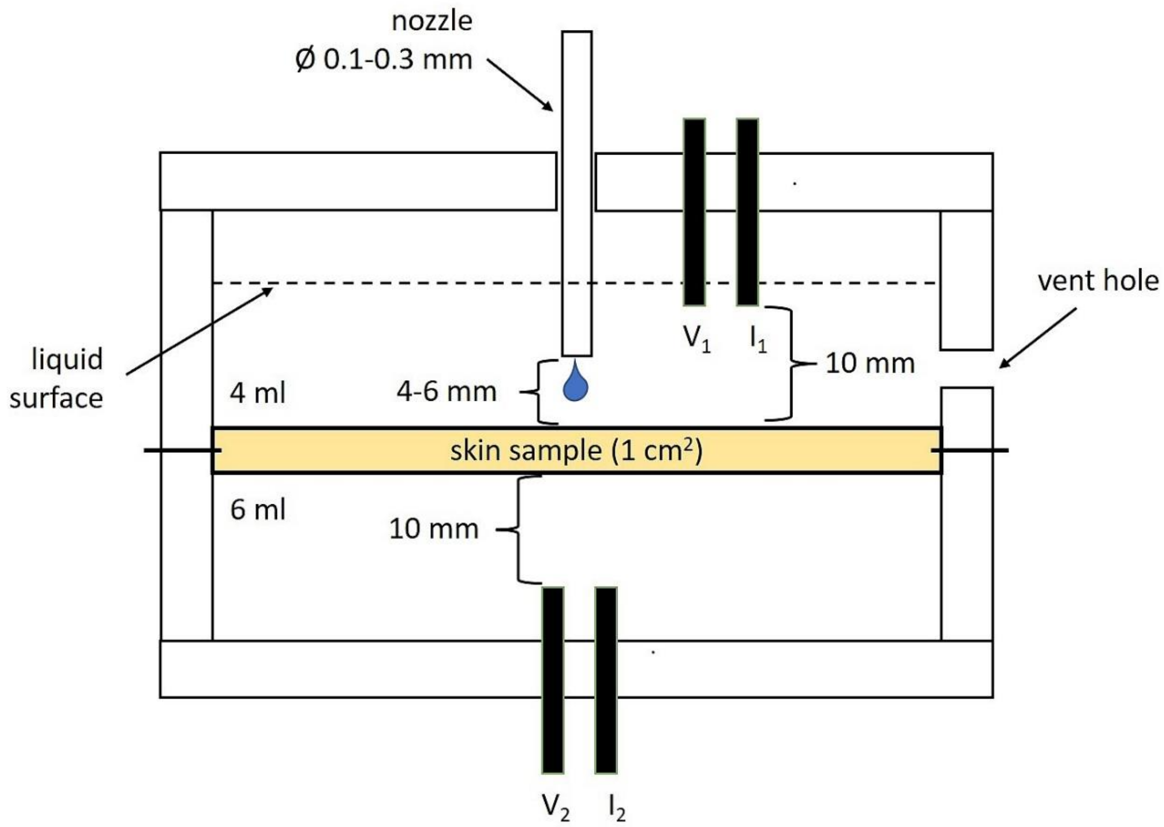

Figure 3S. The scheme of modified Ussing chamber.

Abbreviations: V_1 – a measured electrode, V_2 – a ground electrode, I_1, I_2 - electrodes to pass the current of constant intensity of 10 µA from I_1 side and 10 µA from I_2 side.
